# Supplementary figures and images for: Microbubble dynamics in brain microvessels
Source: PLoS One. 2025 Feb 5;20(2):e0310425. doi: 10.1371/journal.pone.0310425 (PMC11798480; doi:10.1371/journal.pone.0310425)

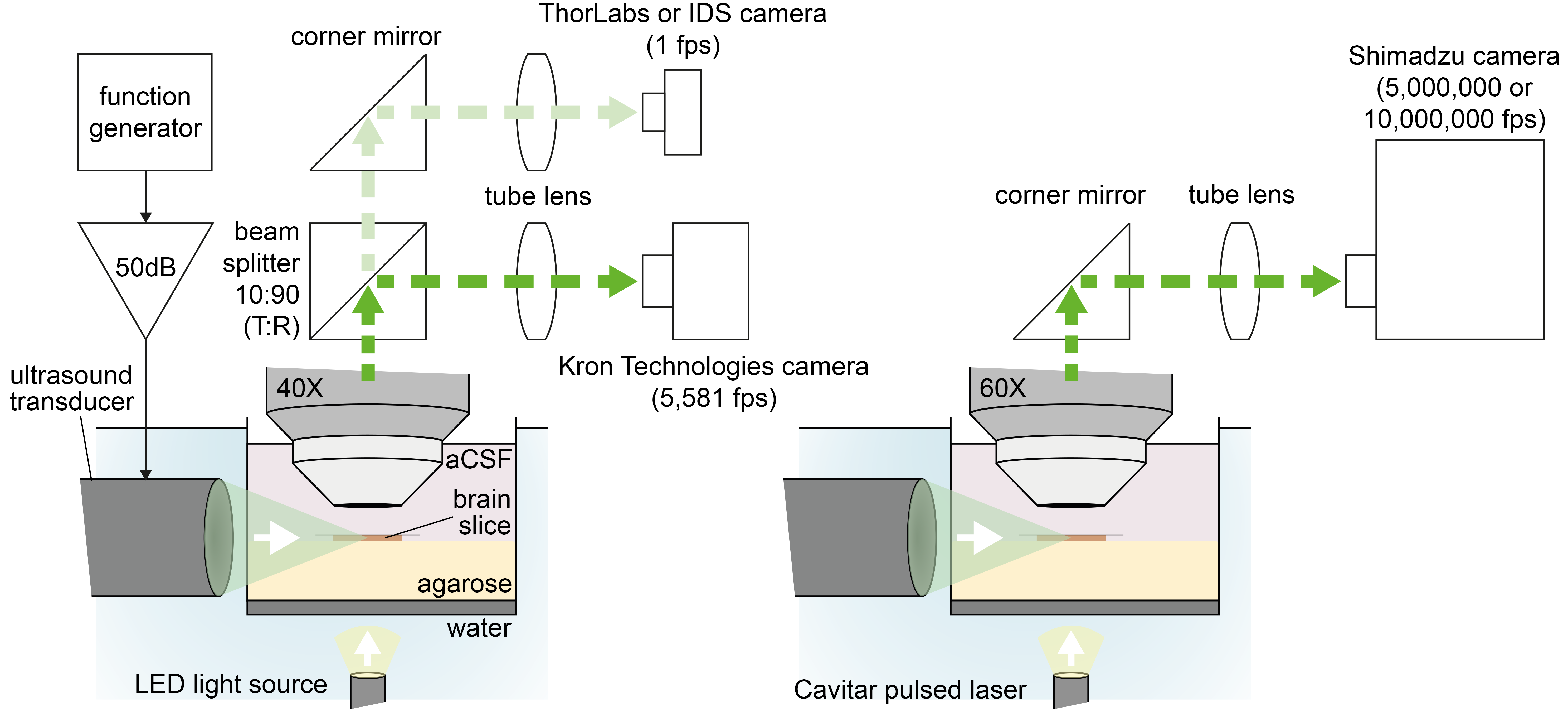

Supplement: S1 Fig — A 250-μm-thick brain slice from a juvenile rat was immersed in artificial cerebrospinal fluid and placed between a light source and an objective. A focused ultrasound transducer emitted sound onto the brain slice, while videos were captured with one of two camera setups. (Left) In order to capture videos on the milliseconds timescale, light from an LED source was transmitted through a 40X objective and guided to a color camera at 1 frame per second (fps) and a high-speed camera at 5,581 fps. (Right) In order to capture videos on the microseconds time scale, light from a Cavitar 10-ns pulsed laser was transmitted through a 60X objective and guided into a Shimadzu camera at 5 or 10 million frames per second. (PNG) [file pone.0310425.s001.png]

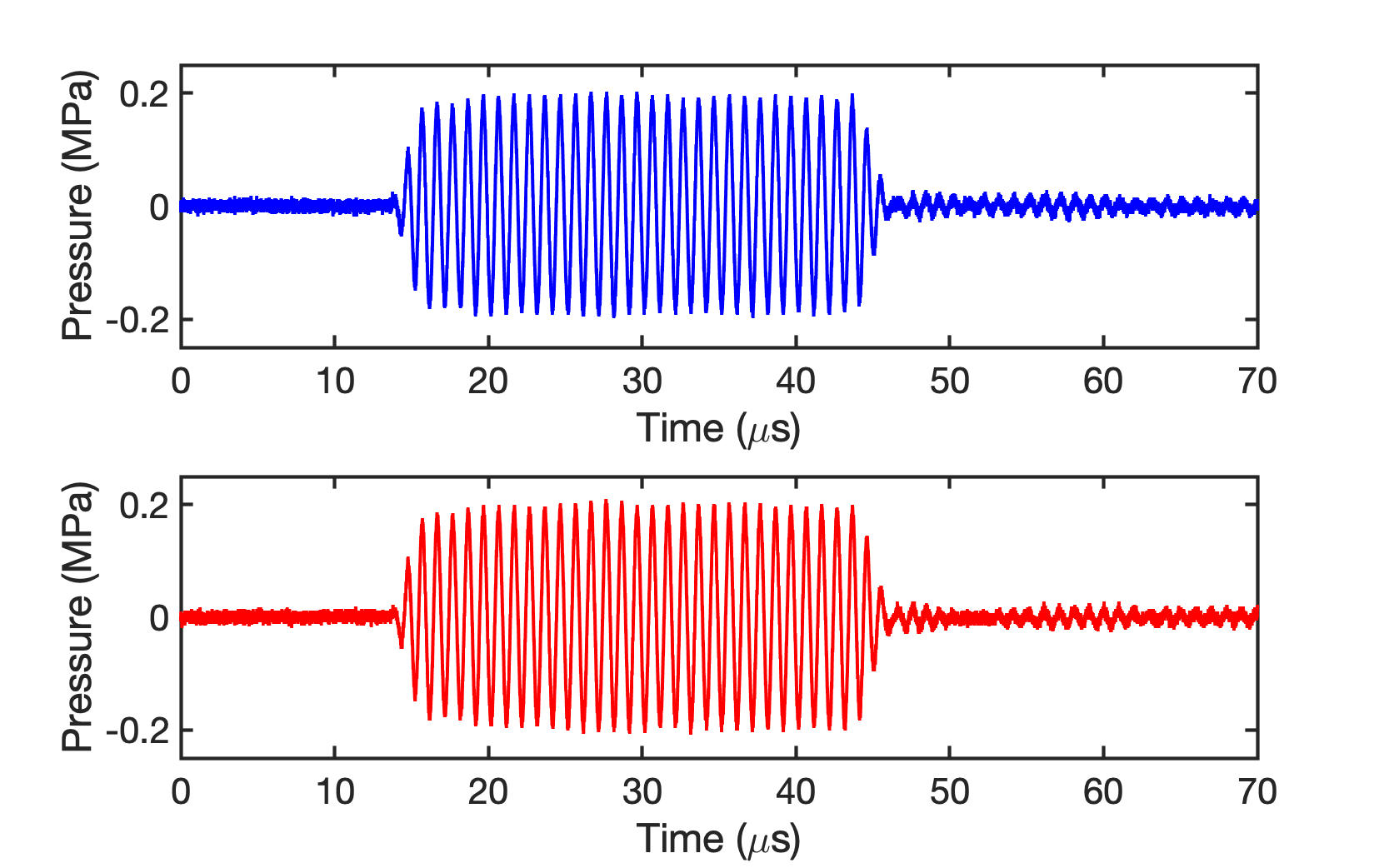

Supplement: S2 Fig — Ultrasound pulses were measured using a needle hydrophone (Top, Blue) without a box present and (Bottom, Red) with the box present. The pressure amplitudes were very similar with or without the box. The ramp-up phase was short and only very low-amplitude ultrasound was present beyond the expected 30-cycle pulse waveform. (PNG) [file pone.0310425.s002.png]

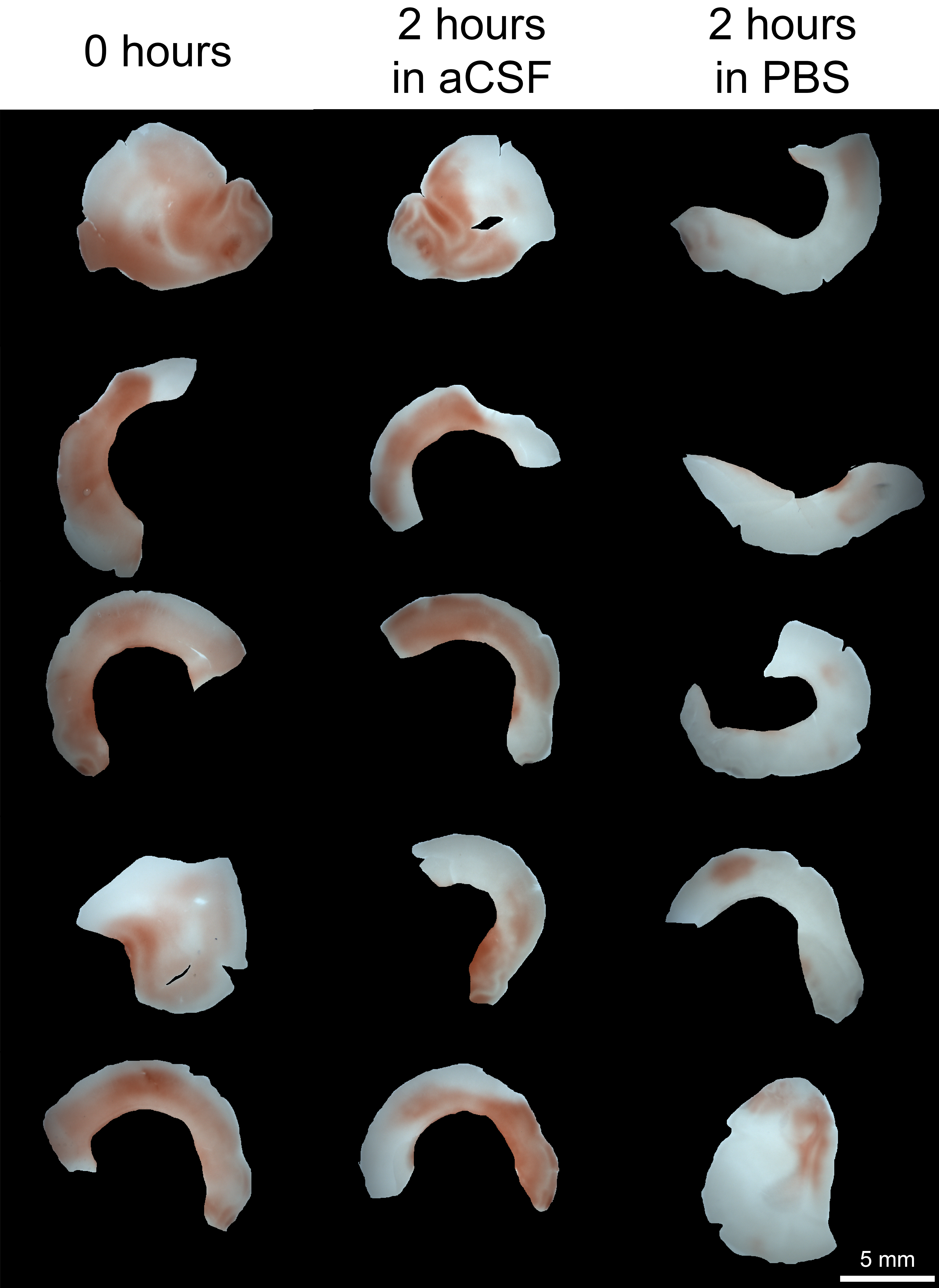

Supplement: S3 Fig — The tissue is stained red when the tissue is undergoing aerobic respiration. The brain slices were stained with TTC (Left) at 0 hours, (Middle) 2 hours in aCSF, and (Right) 2 hours in PBS. The 0-hour time point refers to the time at which a typical experiment started, while the 2-hour time point refers to the time at which a typical experiment ended. (PNG) [file pone.0310425.s003.png]

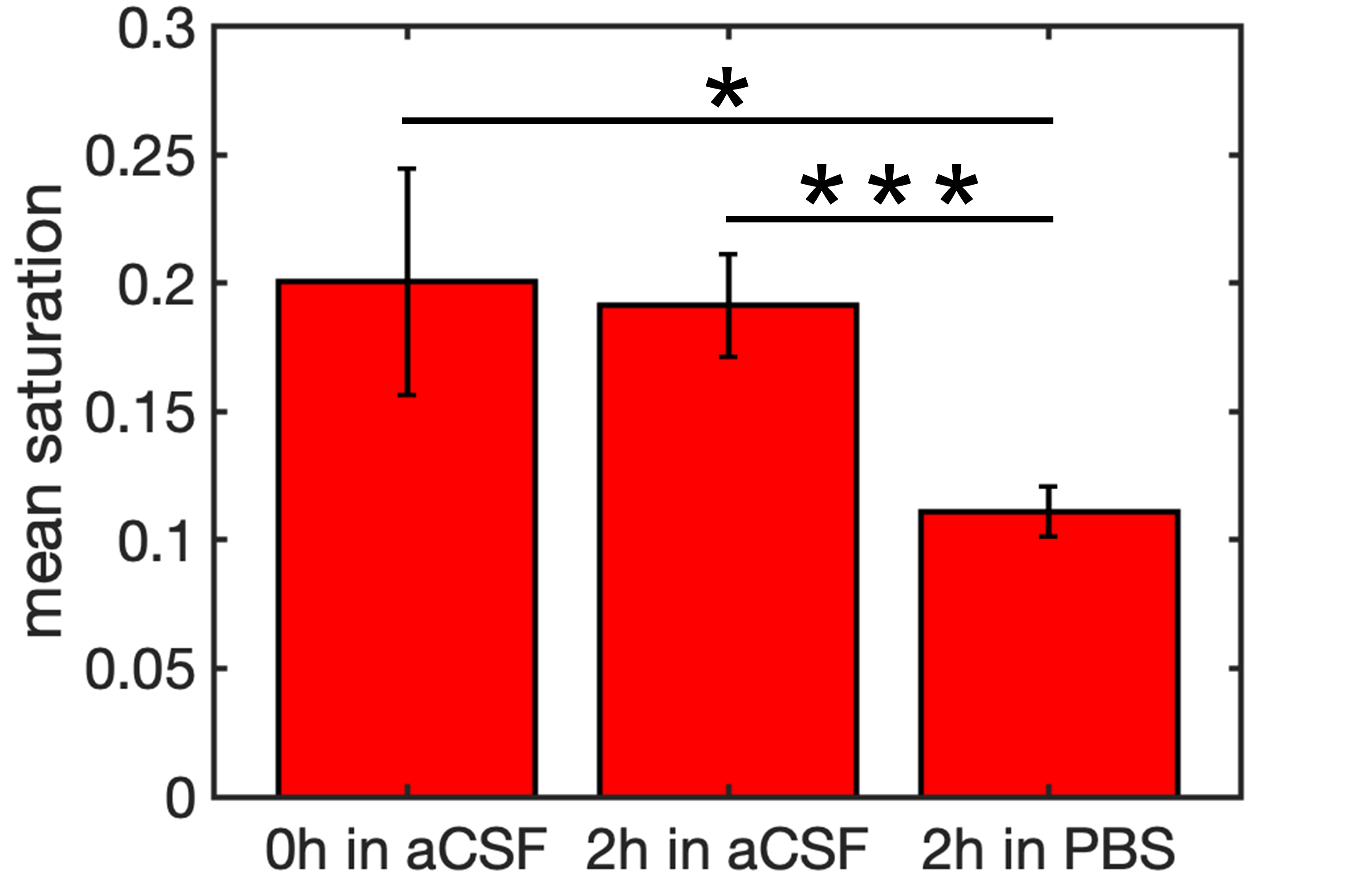

Supplement: S4 Fig — Error bars are standard deviations between slices. * p=0.04, *** p=8.4x10^-5^. (PNG) [file pone.0310425.s004.png]

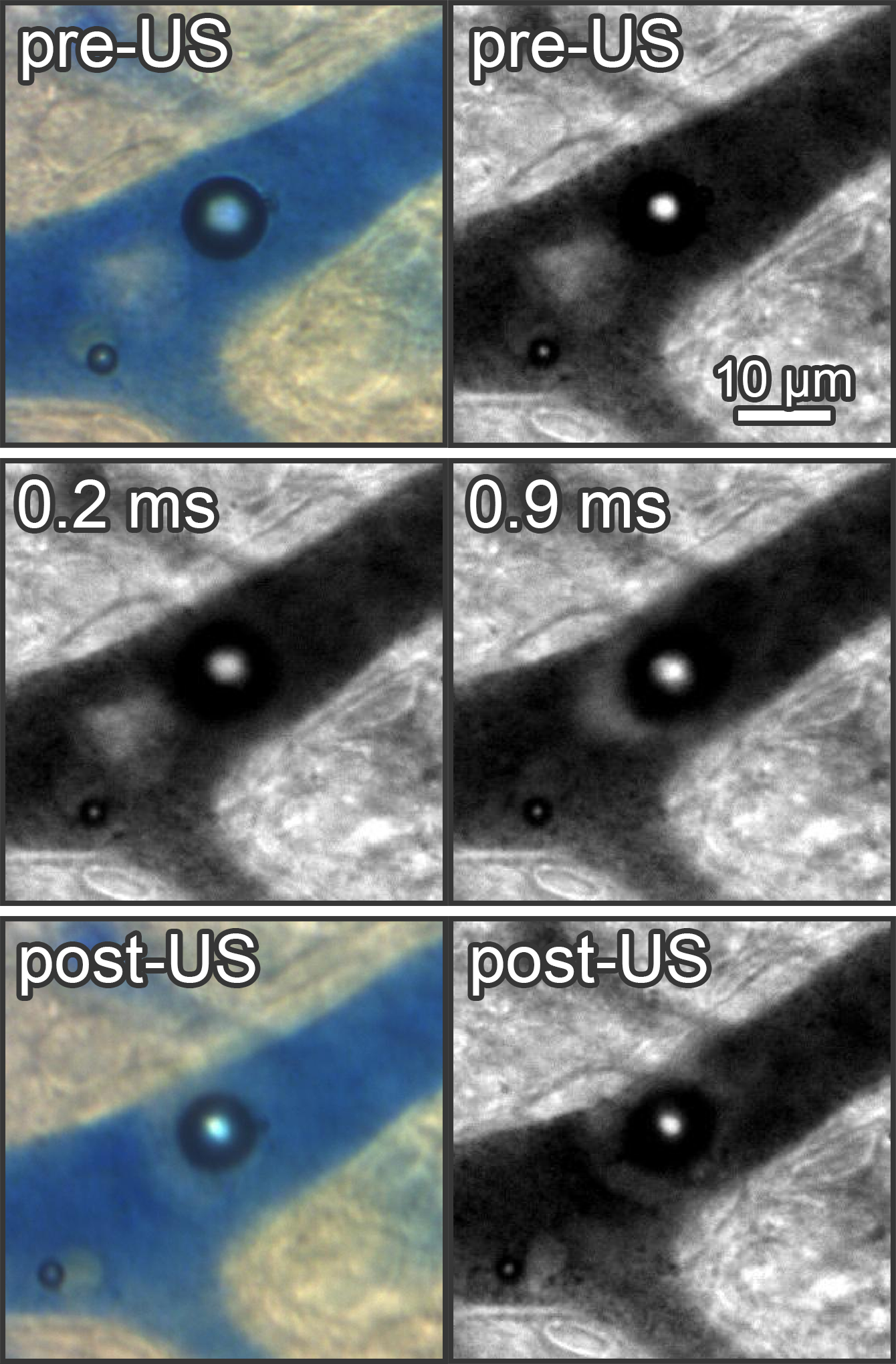

Supplement: S5 Fig — The brain slice was exposed to a 0.4-MPapk-neg, 10-ms pulse and imaged at 5,581 fps. Streaming patterns surrounding the microbubbles were observed during sonication. The region of dye around the bubble was affected by streaming, resulting in a clearly-defined boundary. (PNG) [file pone.0310425.s005.png]
