# Supplementary material for: Microbubble dynamics in brain microvessels
Source: PLoS One. 2025 Feb 5;20(2):e0310425. doi: 10.1371/journal.pone.0310425 (PMC11798480; doi:10.1371/journal.pone.0310425)
Supplement: S1 Table — This data set was used in Fig 5. (DOCX) [file pone.0310425.s014.docx]

**Table S1. Record of microbubbles that did or did not extravasate from microbubbles.** This data set was used in Figure 5.

|  | **Diameter** | **Peak-rarefactional pressure** | **Bubble extravasated? (N: No, Y: Yes, U: Unclear)** | | |
| --- | --- | --- | --- | --- | --- |
| **Sample #** | **(µm)** | **(kPa)** | **Assessor 1** | **Assessor 2** | **Combined** |
| 1 | 6.1 | 200 | N | N | N |
| 2 | 3.9 | 200 | N | N | N |
| 3 | 5.1 | 200 | N | N | N |
| 4 | 3 | 200 | N | N | N |
| 5 | 7.3 | 200 | N | N | N |
| 6 | 5.8 | 200 | N | N | N |
| 7 | 4.3 | 200 | N | N | N |
| 8 | 5.5 | 200 | N | N | N |
| 9 | 4.6 | 200 | N | N | N |
| 10 | 3.6 | 200 | N | N | N |
| 11 | 2.2 | 200 | N | N | N |
| 12 | 4.4 | 200 | N | N | N |
| 13 | 4.5 | 200 | N | N | N |
| 14 | 6.8 | 200 | N | N | N |
| 15 | 2.5 | 200 | N | N | N |
| 16 | 6.5 | 200 | N | N | N |
| 17 | 4.7 | 200 | N | N | N |
| 18 | 3.8 | 400 | N | N | N |
| 19 | 6.1 | 400 | N | N | N |
| 20 | 6.7 | 400 | N | N | N |
| 21 | 5.7 | 400 | N | N | N |
| 22 | 4.1 | 400 | N | N | N |
| 23 | 2 | 400 | N | N | N |
| 24 | 11 | 400 | N | N | N |
| 25 | 5.3 | 400 | N | N | N |
| 26 | 2.5 | 400 | N | N | N |
| 27 | 5.7 | 400 | N | N | N |
| 28 | 4.5 | 400 | N | N | N |
| 29 | 4.1 | 400 | N | N | N |
| 30 | 4.1 | 400 | N | N | N |
| 31 | 3.5 | 400 | N | N | N |
| 32 | 4.1 | 400 | N | N | N |
| 33 | 5.2 | 400 | N | N | N |
| 34 | 3.6 | 400 | N | N | N |
| 35 | 5.4 | 400 | N | Y | U |
| 36 | 3.8 | 400 | N | N | N |
| 37 | 6.1 | 400 | Y | Y | Y |
| 38 | 4.7 | 400 | N | N | N |
| 39 | 3.2 | 400 | N | N | N |
| 40 | 2.9 | 400 | N | N | N |
| 41 | 2.8 | 600 | N | N | N |
| 42 | 2.3 | 600 | N | N | N |
| 43 | 5 | 600 | N | N | N |
| 44 | 5.1 | 600 | Y | N | U |
| 45 | 4.8 | 600 | N | N | N |
| 46 | 4 | 600 | N | N | N |
| 47 | 10.4 | 600 | N | N | N |
| 48 | 4.7 | 600 | N | N | N |
| 49 | 8.4 | 600 | Y | Y | Y |
| 50 | 6 | 600 | Y | Y | Y |
| 51 | 2.2 | 600 | Y | N | U |
| 52 | 3.1 | 600 | N | N | N |
| 53 | 2.3 | 600 | N | N | N |
| 54 | 5.4 | 600 | Y | Y | Y |
| 55 | 3 | 600 | N | N | N |
| 56 | 3.4 | 600 | N | N | N |
| 57 | 3.5 | 600 | N | N | N |
| 58 | 3 | 600 | N | N | N |
| 59 | 4.8 | 600 | Y | Y | Y |
| 60 | 5.2 | 600 | Y | Y | Y |
| 61 | 2.5 | 600 | N | N | U |
| 62 | 4.9 | 800 | Y | Y | Y |
| 63 | 4 | 800 | Y | Y | Y |
| 64 | 5 | 800 | Y | Y | Y |
| 65 | 4 | 800 | Y | Y | Y |
| 66 | 2.6 | 800 | Y | Y | Y |
| 67 | 3.8 | 800 | Y | Y | Y |
| 68 | 7 | 800 | N | N | N |
| 69 | 7.2 | 800 | Y | N | U |
| 70 | 4 | 800 | Y | Y | Y |
| 71 | 7 | 800 | U | N | U |
| 72 | 5.3 | 800 | Y | Y | Y |
| 73 | 2.1 | 800 | U | N | U |
| 74 | 5.5 | 800 | U | N | U |
| 75 | 3.3 | 800 | Y | Y | Y |
| 76 | 3.1 | 800 | U | N | U |
| 77 | 5.7 | 800 | Y | y | Y |
| 78 | 4.9 | 800 | Y | Y | Y |
| 79 | 4.7 | 800 | Y | Y | Y |
| 80 | 4 | 800 | Y | Y | Y |
| 81 | 3.2 | 800 | Y | Y | Y |
| 82 | 8 | 800 | Y | N | U |
